# Supplementary material for: Comparing actuarial and subjective healthy life expectancy estimates: A cross-sectional survey among the general population in Hungary
Source: PLoS One. 2022 Mar 10;17(3):e0264708. doi: 10.1371/journal.pone.0264708 (PMC8912206; doi:10.1371/journal.pone.0264708)

S1 Fig. Distribution of A) subjective life expectancy (sLE) in men, B) sLE in women, C) subjective healthy life expectancy (sHLE) in men and D) sHLE in women

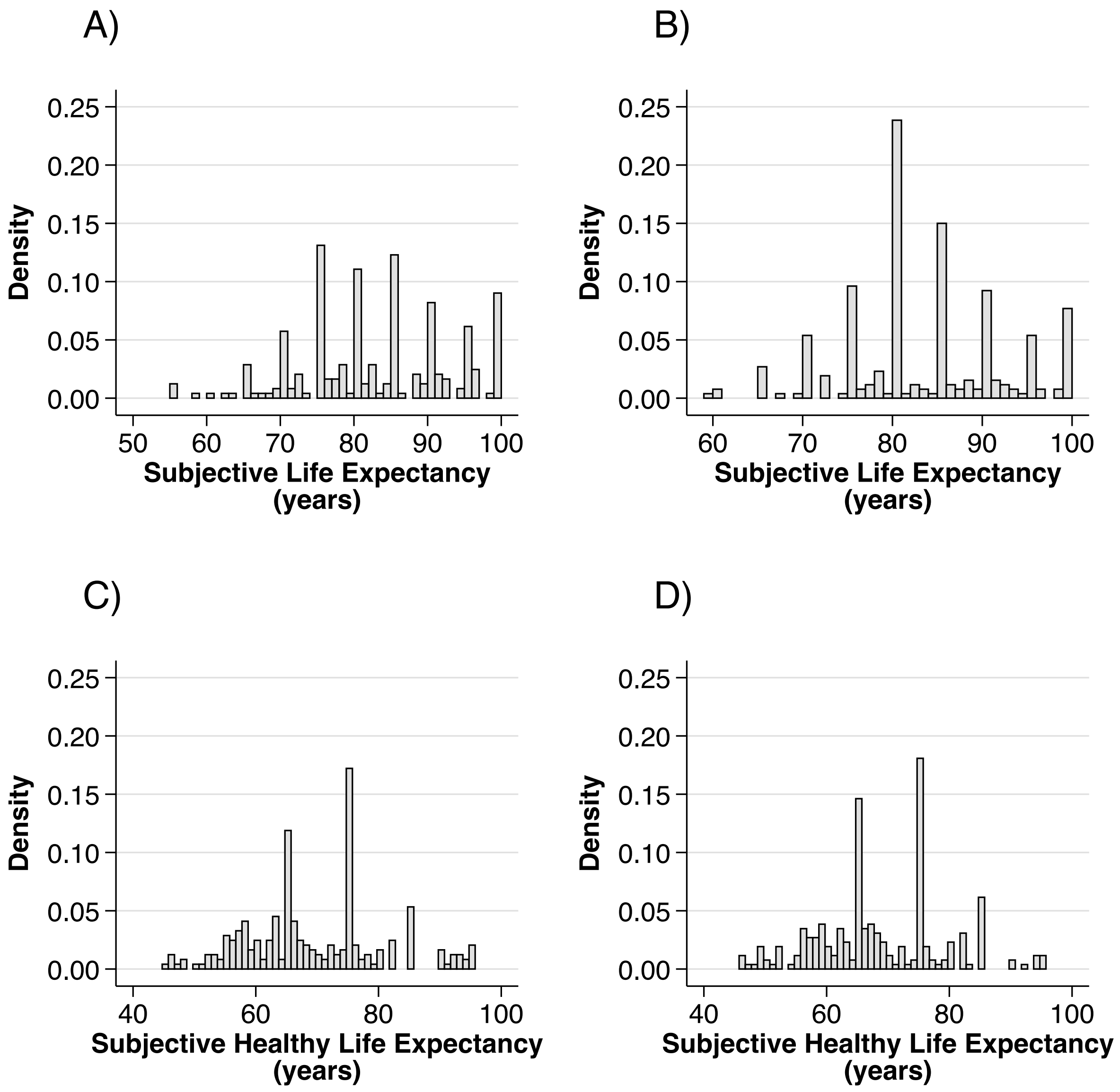

Supplement: S1 Fig — Distribution of A) subjective life expectancy (sLE) in men, B) sLE in women, C) subjective healthy life expectancy (sHLE) in men and D) sHLE in women. (PDF) [file pone.0264708.s001.pdf]
