# Supplementary material for: Comparing actuarial and subjective healthy life expectancy estimates: A cross-sectional survey among the general population in Hungary
Source: PLoS One. 2022 Mar 10;17(3):e0264708. doi: 10.1371/journal.pone.0264708 (PMC8912206; doi:10.1371/journal.pone.0264708)
Supplement: S2 Fig — (PDF) [file pone.0264708.s002.pdf]

S2 Fig. Subjective life expectancy (sLE) versus subjective healthy life expectancy (sHLE) in men and women

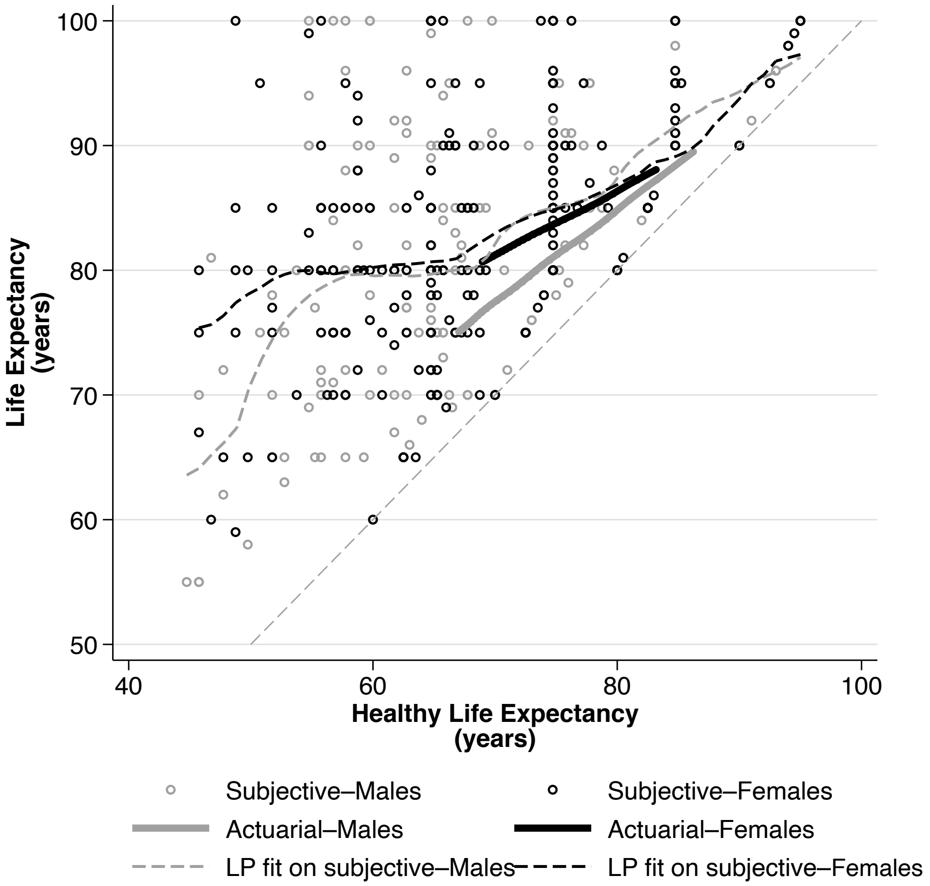

LP: local polynomial
