# Supplementary material for: Comparing actuarial and subjective healthy life expectancy estimates: A cross-sectional survey among the general population in Hungary
Source: PLoS One. 2022 Mar 10;17(3):e0264708. doi: 10.1371/journal.pone.0264708 (PMC8912206; doi:10.1371/journal.pone.0264708)
Supplement: S1 Table — (DOCX) [file pone.0264708.s003.docx]

**S1 Table. Actuarial healthy life expectancy estimates for males by age years**

| Age | | From  100 000 births numbers surviving to age x | | Conditional probability of death* | | Person years lived at age x | | Expected life years | | Life expectancy | | Healthy life years | | Healthy life years by age | | Healthy life expectancy | | Mean HLY by age group | Condi-tional proba-bility of limitations if healthy in previous year |
| --- | --- | --- | --- | --- | --- | --- | --- | --- | --- | --- | --- | --- | --- | --- | --- | --- | --- | --- | --- |
| x | | l_x_ | | q_x_ | | L_x_ | | e_x_ | | LE_x_ | | HLY_i_ | | HLY_x_ | | HLE_x_ | | mHLY_x_ | hq_x_ |
| 50 | 94 285 | | 0.00669 | | 93 970 | | 25.09 | | 75.09 | | 15.51 | | 17.04 | | 67.04 | | 15.51 | | - |
| 51 | 93 655 | | 0.00765 | | 93 297 | | 24.26 | | 75.26 | |  | | 16.26 | | 67.26 | |  | | 0.05185 |
| 52 | 92 938 | | 0.00869 | | 92 535 | | 23.44 | | 75.44 | |  | | 15.50 | | 67.50 | |  | | 0.05418 |
| 53 | 92 131 | | 0.00981 | | 91 679 | | 22.64 | | 75.64 | |  | | 14.75 | | 67.75 | |  | | 0.05675 |
| 54 | 91 227 | | 0.01102 | | 90 724 | | 21.86 | | 75.86 | |  | | 14.01 | | 68.01 | |  | | 0.05960 |
| 55 | 90 222 | | 0.01231 | | 89 666 | | 21.1 | | 76.1 | | 11.85 | | 13.28 | | 68.28 | | 11.92 | | 0.06277 |
| 56 | 89 111 | | 0.01369 | | 88 501 | | 20.35 | | 76.35 | |  | | 12.55 | | 68.55 | |  | | 0.06633 |
| 57 | 87 891 | | 0.01514 | | 87 226 | | 19.63 | | 76.63 | |  | | 11.87 | | 68.87 | |  | | 0.06700 |
| 58 | 86 561 | | 0.01667 | | 85 839 | | 18.92 | | 76.92 | |  | | 11.24 | | 69.24 | |  | | 0.06747 |
| 59 | 85 118 | | 0.0183 | | 84 339 | | 18.24 | | 77.24 | |  | | 10.66 | | 69.66 | |  | | 0.06770 |
| 60 | 83 560 | | 0.02003 | | 82 723 | | 17.57 | | 77.57 | | 9.17 | | 10.12 | | 70.12 | | 9.16 | | 0.06763 |
| 61 | 81 886 | | 0.02192 | | 80 988 | | 16.92 | | 77.92 | |  | | 9.63 | | 70.63 | |  | | 0.06718 |
| 62 | 80 091 | | 0.02395 | | 79 132 | | 16.28 | | 78.28 | |  | | 9.16 | | 71.16 | |  | | 0.07053 |
| 63 | 78 173 | | 0.02605 | | 77 155 | | 15.67 | | 78.67 | |  | | 8.68 | | 71.68 | |  | | 0.07428 |
| 64 | 76 137 | | 0.02813 | | 75 066 | | 15.08 | | 79.08 | |  | | 8.22 | | 72.22 | |  | | 0.07851 |
| 65 | 73 995 | | 0.03012 | | 72 881 | | 14.5 | | 79.5 | | 6.84 | | 7.75 | | 72.75 | | 6.87 | | 0.08332 |
| 66 | 71 766 | | 0.03192 | | 70 621 | | 13.93 | | 79.93 | |  | | 7.28 | | 73.28 | |  | | 0.08884 |
| 67 | 69 476 | | 0.03356 | | 68 310 | | 13.38 | | 80.38 | |  | | 6.84 | | 73.84 | |  | | 0.09013 |
| 68 | 67 144 | | 0.03522 | | 65 962 | | 12.82 | | 80.82 | |  | | 6.44 | | 74.44 | |  | | 0.09096 |
| 69 | 64 779 | | 0.03705 | | 63 579 | | 12.27 | | 81.27 | |  | | 6.06 | | 75.06 | |  | | 0.09114 |
| 70 | 62 379 | | 0.03923 | | 61 156 | | 11.73 | | 81.73 | | 5.16 | | 5.73 | | 75.73 | | 5.11 | | 0.09047 |
| 71 | 59 932 | | 0.04164 | | 58 684 | | 11.19 | | 82.19 | |  | | 5.43 | | 76.43 | |  | | 0.08869 |
| 72 | 57 436 | | 0.04418 | | 56 168 | | 10.65 | | 82.65 | |  | | 5.12 | | 77.12 | |  | | 0.09613 |
| 73 | 54 899 | | 0.047 | | 53 609 | | 10.12 | | 83.12 | |  | | 4.80 | | 77.80 | |  | | 0.10503 |
| 74 | 52 319 | | 0.05026 | | 51 004 | | 9.59 | | 83.59 | |  | | 4.45 | | 78.45 | |  | | 0.11589 |
| 75 | 49 689 | | 0.05413 | | 48 345 | | 9.07 | | 84.07 | | 3.35 | | 4.08 | | 79.08 | | 3.35 | | 0.12941 |
| 76 | 47 000 | | 0.06264 | | 45 528 | | 8.56 | | 84.56 | |  | | 3.68 | | 79.68 | |  | | 0.14673 |
| 77 | 44 056 | | 0.06586 | | 42 605 | | 8.1 | | 85.1 | |  | | 3.32 | | 80.32 | |  | | 0.15357 |
| 78 | 41 154 | | 0.06974 | | 39 719 | | 7.64 | | 85.64 | |  | | 2.99 | | 80.99 | |  | | 0.15971 |
| 79 | 38 284 | | 0.0744 | | 36 860 | | 7.18 | | 86.18 | |  | | 2.69 | | 81.69 | |  | | 0.16420 |
| 80 | 35 435 | | 0.08 | | 34 018 | | 6.71 | | 86.71 | | 1.98 | | 2.42 | | 82.42 | | 1.97 | | 0.16551 |
| 81 | 32 601 | | 0.08671 | | 31 187 | | 6.25 | | 87.25 | |  | | 2.21 | | 83.21 | |  | | 0.16126 |
| 82 | 29 774 | | 0.09474 | | 28 364 | | 5.8 | | 87.8 | |  | | 1.99 | | 83.99 | |  | | 0.17869 |
| 83 | 26 953 | | 0.10435 | | 25 547 | | 5.35 | | 88.35 | |  | | 1.75 | | 84.75 | |  | | 0.20104 |
| 84 | 24 141 | | 0.11581 | | 22 743 | | 4.92 | | 88.92 | |  | | 1.50 | | 85.50 | |  | | 0.23095 |
| 85 | 21 345 | | 0.12945 | | 19 964 | | 4.5 | | 89.5 | | 1.05 | | 1.24 | | 86.24 | | - | | 0.27341 |

*Life table of Hungary 2019, source: Hungarian Central Statistical Office
