# Supplementary material for: Comparing actuarial and subjective healthy life expectancy estimates: A cross-sectional survey among the general population in Hungary
Source: PLoS One. 2022 Mar 10;17(3):e0264708. doi: 10.1371/journal.pone.0264708 (PMC8912206; doi:10.1371/journal.pone.0264708)
Supplement: S2 Table — (DOCX) [file pone.0264708.s004.docx]

**S2 Table. Actuarial healthy life expectancy estimates for females by age years**

| Age | From  100 000 births numbers surviving to age x | Conditional probability of death* | Person years lived at age x | Expected life years | Life expectancy | Healthy life years | Healthy life years by age | Healthy life expectancy | Mean HLY by age group | Condi-tional proba-bility of limitations if healthy in previous year |
| --- | --- | --- | --- | --- | --- | --- | --- | --- | --- | --- |
| x | l_x_ | q_x_ | L_x_ | e_x_ | LE_x_ | HLY_i_ | HLY_x_ | HLE_x_ | mHLY_x_ | 1-hq_x_ |
| 50 | 97 079 | 0.00283 | 96 941 | 30.69 | 80.69 | 17.34 | 18.96 | 68.96 | 17.34 | - |
| 51 | 96 804 | 0.00326 | 96 647 | 29.77 | 80.77 |  | 18.13 | 69.13 |  | 0.04649 |
| 52 | 96 489 | 0.00375 | 96 308 | 28.87 | 80.87 |  | 17.32 | 69.32 |  | 0.04784 |
| 53 | 96 127 | 0.0043 | 95 920 | 27.98 | 80.98 |  | 16.53 | 69.53 |  | 0.04927 |
| 54 | 95 713 | 0.00488 | 95 480 | 27.1 | 81.1 |  | 15.76 | 69.76 |  | 0.05080 |
| 55 | 95 246 | 0.00549 | 94 985 | 26.23 | 81.23 | 13.57 | 15.00 | 70.00 | 13.57 | 0.05245 |
| 56 | 94 724 | 0.00612 | 94 434 | 25.37 | 81.37 |  | 14.27 | 70.27 |  | 0.05421 |
| 57 | 94 144 | 0.00677 | 93 825 | 24.52 | 81.52 |  | 13.55 | 70.55 |  | 0.05595 |
| 58 | 93 507 | 0.00747 | 93 158 | 23.68 | 81.68 |  | 12.86 | 70.86 |  | 0.05782 |
| 59 | 92 808 | 0.00819 | 92 428 | 22.86 | 81.86 |  | 12.18 | 71.18 |  | 0.05983 |
| 60 | 92 048 | 0.00895 | 91 636 | 22.04 | 82.04 | 10.27 | 11.52 | 71.52 | 10.29 | 0.06200 |
| 61 | 91 224 | 0.00974 | 90 780 | 21.24 | 82.24 |  | 10.87 | 71.87 |  | 0.06435 |
| 62 | 90 336 | 0.01055 | 89 859 | 20.44 | 82.44 |  | 10.26 | 72.26 |  | 0.06576 |
| 63 | 89 383 | 0.0114 | 88 874 | 19.65 | 82.65 |  | 9.67 | 72.67 |  | 0.06716 |
| 64 | 88 364 | 0.0123 | 87 821 | 18.88 | 82.88 |  | 9.11 | 73.11 |  | 0.06854 |
| 65 | 87 277 | 0.01328 | 86 697 | 18.1 | 83.1 | 7.61 | 8.58 | 73.58 | 7.60 | 0.06988 |
| 66 | 86 118 | 0.01428 | 85 503 | 17.34 | 83.34 |  | 8.08 | 74.08 |  | 0.07114 |
| 67 | 84 888 | 0.01527 | 84 240 | 16.59 | 83.59 |  | 7.59 | 74.59 |  | 0.07386 |
| 68 | 83 592 | 0.01636 | 82 908 | 15.84 | 83.84 |  | 7.12 | 75.12 |  | 0.07681 |
| 69 | 82 224 | 0.01763 | 81 499 | 15.09 | 84.09 |  | 6.66 | 75.66 |  | 0.08001 |
| 70 | 80 774 | 0.01919 | 80 000 | 14.35 | 84.35 | 5.38 | 6.21 | 76.21 | 5.35 | 0.08350 |
| 71 | 79 225 | 0.02087 | 78 398 | 13.62 | 84.62 |  | 5.78 | 76.78 |  | 0.08733 |
| 72 | 77 571 | 0.02261 | 76 694 | 12.9 | 84.9 |  | 5.35 | 77.35 |  | 0.09360 |
| 73 | 75 817 | 0.02465 | 74 883 | 12.19 | 85.19 |  | 4.92 | 77.92 |  | 0.10097 |
| 74 | 73 948 | 0.02721 | 72 943 | 11.48 | 85.48 |  | 4.49 | 78.49 |  | 0.10975 |
| 75 | 71 936 | 0.03051 | 70 839 | 10.79 | 85.79 | 3.29 | 4.06 | 79.06 | 3.29 | 0.12041 |
| 76 | 69 741 | 0.03659 | 68 466 | 10.12 | 86.12 |  | 3.63 | 79.63 |  | 0.13363 |
| 77 | 67 190 | 0.04012 | 65 842 | 9.48 | 86.48 |  | 3.25 | 80.25 |  | 0.13763 |
| 78 | 64 494 | 0.04434 | 63 064 | 8.86 | 86.86 |  | 2.91 | 80.91 |  | 0.14032 |
| 79 | 61 634 | 0.04936 | 60 113 | 8.24 | 87.24 |  | 2.62 | 81.62 |  | 0.14082 |
| 80 | 58 592 | 0.05534 | 56 971 | 7.65 | 87.65 | 1.97 | 2.37 | 82.37 | 1.98 | 0.13782 |
| 81 | 55 350 | 0.06243 | 53 622 | 7.07 | 88.07 |  | 2.19 | 83.19 |  | 0.12960 |
| 82 | 51 894 | 0.07085 | 50 056 | 6.5 | 88.5 |  | 1.99 | 83.99 |  | 0.14526 |
| 83 | 48 217 | 0.08083 | 46 268 | 5.96 | 88.96 |  | 1.79 | 84.79 |  | 0.16568 |
| 84 | 44 320 | 0.09263 | 42 267 | 5.44 | 89.44 |  | 1.57 | 85.57 |  | 0.19347 |
| 85 | 40 214 | 0.10655 | 38 072 | 4.94 | 89.94 | 1.26 | 1.33 | 86.33 | - | 0.23355 |

*Life table of Hungary 2019, source: Hungarian Central Statistical Office
