# Supplementary material for: Comparing actuarial and subjective healthy life expectancy estimates: A cross-sectional survey among the general population in Hungary
Source: PLoS One. 2022 Mar 10;17(3):e0264708. doi: 10.1371/journal.pone.0264708 (PMC8912206; doi:10.1371/journal.pone.0264708)

S1 Appendix. Details of survey items on subjective expectations and applied data transformations

A) Inquiring subjective life expectancy (sLE) using the point-estimate method

"According to your opinion, until what age will you live?"

B) Inquiring future health expectations (adapted Global Activity Limitation Indicator, GALI)

Question item: *"According to your opinion, will you be limited for at least 6 months in activities people usually do because of health problems, and if yes, to what extent?*"

The response options for the relevant ages (60, 70, 80, 90):
"At the age of ..., I will be:
- *not limited at all*
- *limited but not severely*
- *severely limited"*

C) Imputing subjective healthy life expectancy (sHLE) point estimates

Point estimates of sHLE were imputed according to respondents' current (at the time of survey) and future GALI responses (at ages 60,70,80 and 90) and sLE according to Eq. 1,

$$sHLE = \begin{cases} type\ 1\text{:} \dfrac{a_h + a_l - 0.5}{2}\ if\ \exists\ a_h\ \wedge \exists\ a_l \wedge sLE > a_l \\ type\ 2\text{:} \dfrac{a_h + sLE}{2}\ if\ \exists\ a_h\ \wedge \nexists\ a_l \wedge sLE < a_h + 10 \\ type\ 3\text{:} \dfrac{x + a_l - 0.5}{2}\ if\ \nexists\ a_h\ \wedge \exists\ a_l\ \wedge\ sLE > a_l\ \wedge\ H = h \\ type\ 4\text{:} \dfrac{x + sLE}{2}\ if\ \nexists\ a_h\ \wedge\ 0 \le sLE < x + 10\ \wedge\ H = h \\ type\ 5\text{:} x - 5.25\ if\ \nexists\ a_h\ \wedge\ H = l \end{cases} \tag{1}$$

Notation:

x: respondents' age
sLE: the point estimate of subjective life expectancy
$a_h \in \{60,70,80,90\}$: the greatest future (healthy) age without expected limitation
$\exists\ a_h$: future healthy age expectation is reported (e.g., 60 years)
$\nexists\ a_h$: future healthy age expectation is not reported (e.g., the respondent expects disability from 60 years of age)
$a_l \in \{60,70,80,90\}$: the earliest expected future age with limitation ($x \le a_l$)
$\exists\ a_l$: earliest future age with expected limitation reported (e.g., 70 years)
$\nexists\ a_l$: earliest future age with expected limitation is not reported (e.g., no limitations at 70 years, expected death at 75 years)
$H \in \{h, l\}$: current health measured by GALI ($H = h$: no current limitation, $H = l$: any current limitation)

sHLE point estimate imputation types:

Type 1: both a_h and a_l are provided by the respondent, *sHLE* is the mean of possible ages without limitation

Type 2: a_h and sLE are provided by the respondent, *sHLE* is the mean of possible ages without limitation until expected death

(Assumption: if a_h was provided, then we assumed that respondents' current limitations were temporary and would subside.)

Type 3: no current limitation at age x, the future expectation is limitation (a_l) with no future healthy age reported (a_h): sHLE is the weighted mean of all possible ages without limitation between x and a_l

Type 4: no current limitation at age x, death is expected earlier than future ages with (a_l) or without limitation (a_h): sHLE is the weighted mean of all possible ages without limitation between x and *sLE.*

Type 5: current limitation at age x, no future age expected without limitation: sHLE is the weighted mean of possible healthy ages before age x. (Assumption: the limitation commenced any time during the past 10 years).

Further assumptions: the imagination of immediate death in a healthy person is possible; the GALI suggests 6 months limited period before the commencement of expected future limitations. (Hence the *-0.5* adjustment in the formulas of type 1-, 3- and 5 estimates.)

D) *Examples for subjective healthy life expectancy (sHLE) imputation and incongruent response patterns*

**Type**	***Age (x)***	H_x	H_{60}	H_{70}	H_{80}	H_{90}	a_h	a_l	***sLE***	***sHLE***
1	56	*h*	*h*	*h*	*l*	*l*	70	80	88	74.75
	67	*l*	-	*h*	*h*	*h*	90	-	96	93
2	53	*h*	*h*	*h*	-	-	70	-	78	74
	64	*l*	-	*h*	*h*	-	80	-	86	83
3	58	*h*	*l*	*l*	*l*	-	-	60	80	58.75
	79	*h*	-	-	*l*	-	-	80	85	79.25
4	62	*h*	*l*	-	-	-	-	60	65	63.5
	80	*h*	-	-	*l*	-	-	-	81	80.5
5	51	*l*	*l*	*l*	-	-	-	-	75	45.75
	78	*l*	-	-	*l*	*l*	-	-	90	72.75
Incongruent	53	*h*	*h*	*l*	*h*	*l*	-	-	95	-
Incongruent	69	*l*	-	*l*	*h*	*l*	-	-	90	-

H_x: current limitation at age x, measured with the Global Activity Limitation Indicator (GALI), H_{60}-H_{90} future health expectations measured by adapted GALI; *h*: no limitation, *l*: any limitation, a_h: highest future age without expected limitation, a_l: lowest future age with expected limitation, *sLE*: subjective life expectancy*;* *sHLE*: subjective healthy life expectancy

E) Subjective healthy life expectancy (sHLE) by imputation types

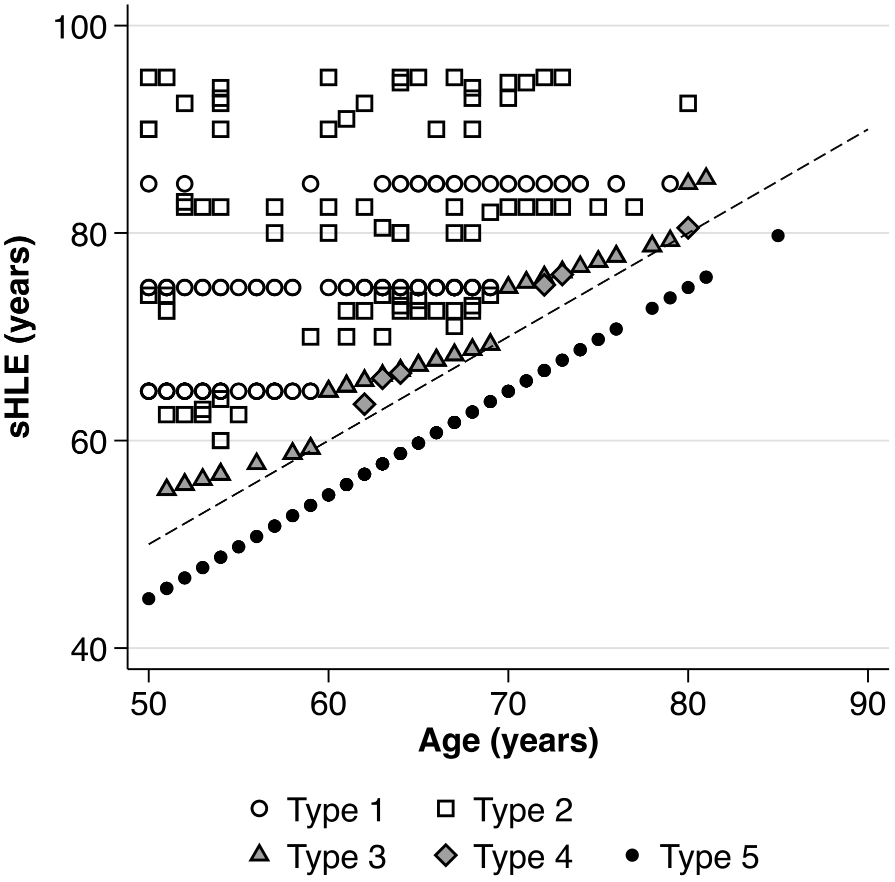

Supplement: S1 Appendix — (PDF) [file pone.0264708.s005.pdf]
