# Supplementary material for: Comparing actuarial and subjective healthy life expectancy estimates: A cross-sectional survey among the general population in Hungary
Source: PLoS One. 2022 Mar 10;17(3):e0264708. doi: 10.1371/journal.pone.0264708 (PMC8912206; doi:10.1371/journal.pone.0264708)
Supplement: S2 Appendix — (PDF) [file pone.0264708.s006.pdf]

S2 Appendix. Estimating actuarial healthy life years (HLY) for each age year from abridged HLY tables

Notation:

HLY_i: actuarial HLY for 5-year age groups i ($i \in \{50-54, 55-59, \ldots 80-84\}$), reported the Hungarian Central Statistical Office (HCSO)

HLY'_x: intermediate estimates of actuarial healthy life years for age *x*

HLY_x: estimated actuarial healthy life years for age *x* ($x \in \{50, 51, \ldots 84\}$)

HLY_{85}: estimated actuarial healthy life years for 85 years of age

$mHLY_{xi}$: mean HLY_x in age group *i*

TH'_x: intermediate estimates of cumulative healthy years lived from year *x*

TH_x: estimated cumulative healthy years lived from year *x*

l_x: the number of persons who reached age *x,* reported by the HCSO

H'_x: intermediate estimates of healthy years for the cohort aged *x*

H_x: smoothed estimates of healthy years for the cohort aged *x*

hq'_x: intermediate conditional probability estimates of becoming limited in age *x* given being healthy in year *x-1.* ($hq'_x < 1$)

hq_x: conditional probability estimates of becoming limited in age *x* given being healthy in year *x-1.* ($0 < hq_x < 1$)

Estimation procedure of HLY_x:

Step 1: we predicted HLY'_x by fitting a piecewise linear regression on HLY_i for age groups *i* ($i \in \{50-54, 55-59, \ldots 80-84\}$) for both sexes using a spline of age as predictor with knots in age-group boundaries (e.g., 50, 55,80, 85).

Healthy life year estimates for age years 50-84 in A) men and B) women

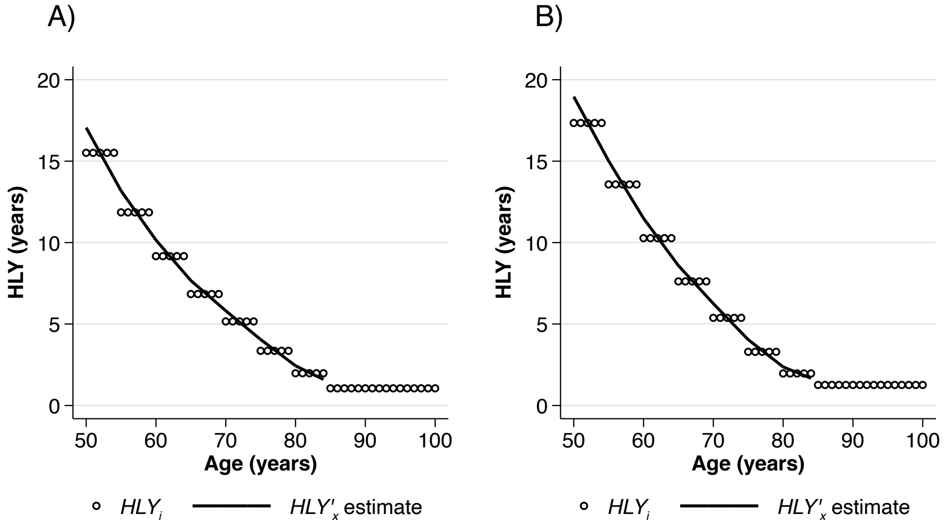

Step 2: by reversing calculations of the Sullivan method, from l_x of the life table, and HLY'_x from Step 1, we calculated TH'_x using the formula $TH'_x = l_x * HLY'_x$.

Step 3: we calculated H'_x for each age *x* via $H'_x = TH'_x - TH'_{x+1}$.

Step 4: we obtained hq'_x by using the formula $hq'_x = 1 - \frac{H'_x}{H'_{x-1}}$. Since we identified negative hq'_x values, we considered H'_x estimates implausible.

Step 5: we estimated H_x by smoothing H'_x via a piecevise regression using the age spline of Step 1.

Cumulative cohort healthy life years (H_x) estimates for age years 50-84 in A) men and B) women

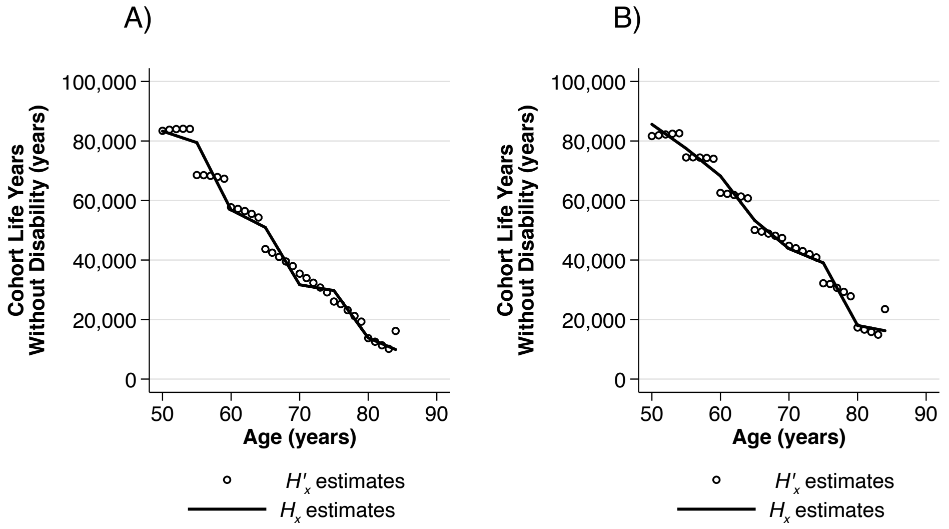

Step 6: we obtained TH_x by the formula $TH_x = \sum_{n \geq x} H_n$.

Step 7: we obtained hq_x by using the formula $hq_x = 1 - \frac{TH_x}{TH_{x-1}}$ and verified the plausibility of $H_x$estimates by observing for each hq_x that $0 < hq_x < 1$.

Proportion becoming limited (hq_x), if being healthy in preceding year in A) men and B) women

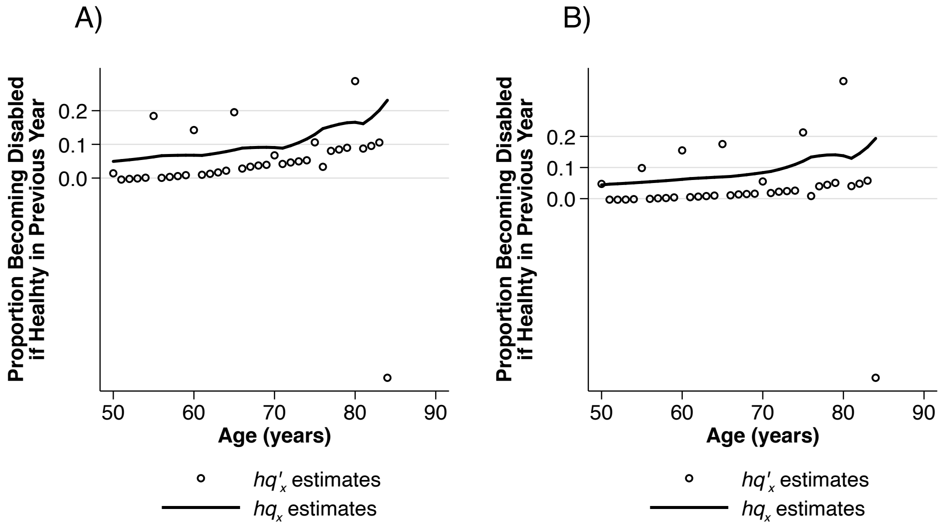

Step 8: we obtained HLY_x via the Sullivan calculation using H_x from Step 5: $HLY_x = \frac{\sum_{n \geq x} H_n}{l_x}$.

Step 9: we estimated HLY_{85} as follows: since HLY_i represents the average healthy life expectancy for the entire 85+ age-group (HLY_{85+}), assuming that $HLY_{85} = HLY_{85+}$ would underestimate healthy life expectancies in all ages x≥85 years. Therefore, we used $HLY_{85} = HLY_{85+} + d$ in the model and determined d via numeric optimisation so that the squared difference between HLY_i and $mHLY_{xi}$ was set to minimum.

Healthy life years by 5-year age groups (HLY$_i$) and means of yearly healthy life year estimates by 5-year age groups (mHLY$_{xi}$) in A) men and B) women

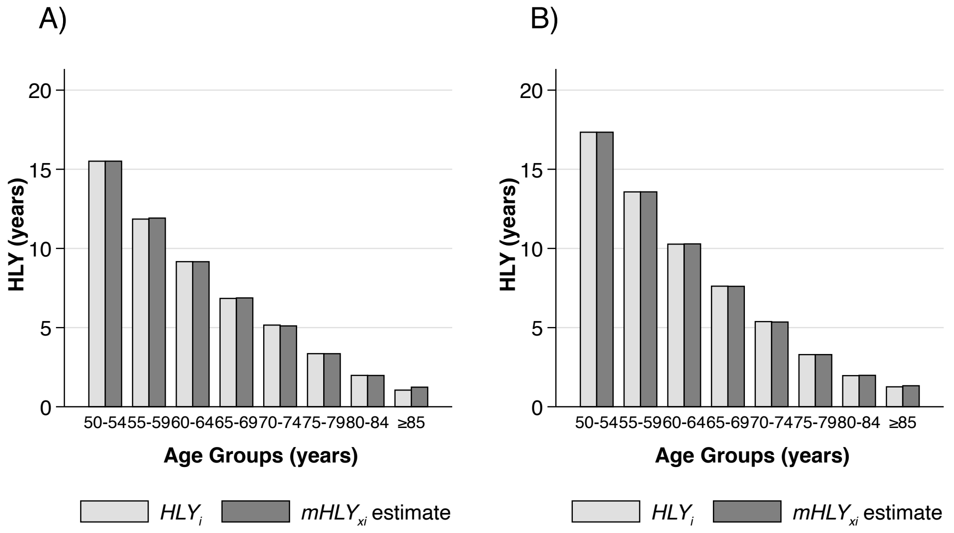

Step 10: we obtained $HLE_x = HLY_x + x$ for all ages x from 50 to 85 years.
